# An Estimate of the Amount of Geological CO_2_ Storage over the Period of 1996–2020

**DOI:** 10.1021/acs.estlett.2c00296

**Published:** 2022-07-19

**Authors:** Yuting Zhang, Christopher Jackson, Samuel Krevor

**Affiliations:** † Royal School of Mines, Imperial College London, Prince Consort Road, South Kensington, London SW7 2BP, U.K.; § Department of Earth Science and Engineering, Imperial College London, London SW7 2BP, U.K.

**Keywords:** CCS, carbon storage, energy, climate change mitigation, CCS statistics

## Abstract

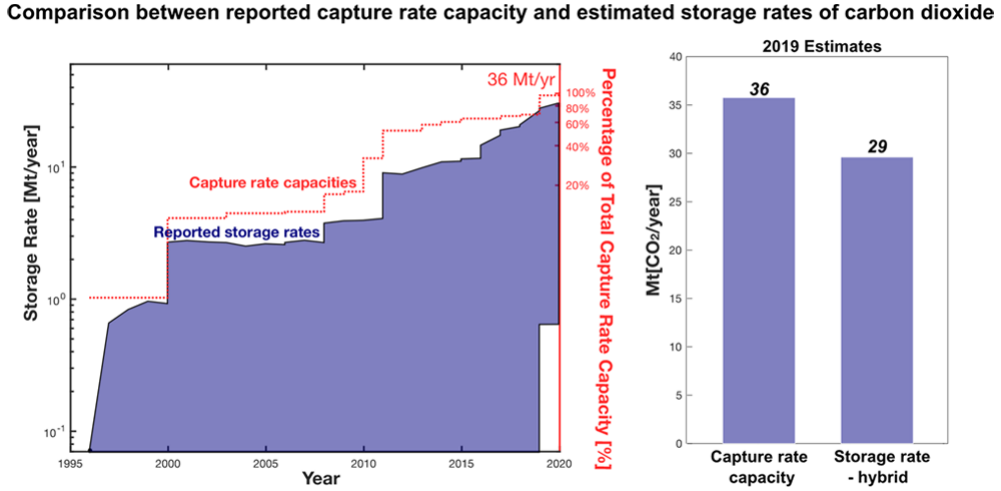

The climate impact of carbon capture and storage depends on how much CO_2_ is stored underground, yet databases of industrial-scale projects report capture capacity as a measure of project size. We review publicly available sources to estimate the amount of CO_2_ that has been stored by facilities since 1996. We organize these sources into three categories corresponding to the associated degree of assurance: (1) legal assurance, (2) quality assurance through auditing, and (3) no assurance. Data were found for 20 facilities, with an aggregate capture capacity of 36 Mt of CO_2_ year^–1^. Combining data from all categories, we estimate that 29 Mt of CO_2_ was geologically stored in 2019 and there was cumulative storage of 197 Mt over the period of 1996–2020. These are climate relevant scales commensurate with recent cumulative and ongoing emissions impacts of renewables in some markets, e.g., solar photovoltaics in the United States. The widely used capture capacity is in aggregate 19–30% higher than storage rates and is not a good proxy for estimating storage volumes. However, the discrepancy is project-specific and not always a reflection of project performance. This work provides a snapshot of storage amounts and highlights the need for uniform reporting on capture and storage rates with quality assurance.

## 1 Introduction

Modeled energy systems development pathways limiting global warming to <2 °C suggest that rapid upscaling of carbon capture and storage (CCS) with global injection rates reaching 5–10 Gt of CO_2_ year^–1^ by 2050 may be required.^1^ Due to the importance of CCS in modeled climate mitigation pathways, the feasibility of achieving these rates by midcentury is central to our understanding of the potential to avoid dangerous climate change. With increasing numbers of industry-scale storage projects operating around the world, data through which project performance, and scale-up potential, may be evaluated are becoming available.

The most centralized and up-to-date information comes from the annual reports and database of the Global CCS Institute (GCCSI).^2^ Similar data sets were produced in the recent past by the MIT Carbon Capture and Sequestration Technologies Program^3^ and the National Energy Technology Laboratory (NETL).^4^ However, they stopped updating in 2016 and 2019, respectively. Additionally, there are several Web sites compiling lists of active CCS projects.^5,6^ In many cases, the GCSSI is used as the primary source of these compilations.^3−6^ The measure used across databases to describe the size of projects is the capture capacity reported in megatonnes per annum (Mtpa). As of 2021, the global capture capacity was estimated at 40 Mt of CO_2_ year^–1^ from 26 operational CCS facilities.^2,7−9^

Despite this reporting, there are information gaps that present challenges to quantifying the current state of CCS. There is no set definition of capture capacity. It appears to take on various meanings among projects, including an aspirational target, a maximum based on capture facility design, and a capture rate achieved in a particular year. Actual rates of capture, transport, and storage are not centrally reported. This information is necessary for the evaluation of the climate change mitigation impact of existing operations. Tracking amounts of CO_2_ captured, transported, and stored can help to identify factors arising throughout a CCS chain. Variations in the performance of industry-scale CCS may also help us to understand and mitigate the range of issues affecting the performance of projects.

In this study, we investigate publicly available information on CO_2_ storage rates for industrial-scale CCS projects since 1996, the first year of injection for the Sleipner project in Norway. We first classify the data sources and review how current statistics are reported. From this, we compile a global CO_2_ storage database and estimate the amount of CO_2_ that has been captured and geologically stored. We analyze discrepancies between estimated storage rates and the more widely reported capture capacity. Finally, we provide recommendations for future reporting.

## 2 Materials and Methods

### 2.1 Project Selection

We use the database of the GCCSI, cross-checked against other databases where possible, to identify industrial-scale projects.^2^ Of the 26 operational carbon capture facilities listed in GCCSI, we estimate captured and stored amounts for 20 of these projects, representing 93% of the existing global operational capture capacity. The 2020 GCCSI database provides the name of the capture facility,^2^ so we first identify the associated storage operators and sites for each capture project by performing a review of online resources using capture facility names as initial keywords in search engines. We find relevant Web pages that provide descriptions of the capture and storage projects, i.e., project Web sites, CCS databases, or operator’s Web sites.^3−6^ We provide the final data references used in the sources column in Tables 3–16 of the Supporting Information. In our database, 14 projects are enhanced oil recovery (EOR) in which the CO_2_ is injected into depleted oil reservoirs to recover additional oil and six projects are storing CO_2_ in deep saline aquifers for dedicated long-term geological storage.^2,8^ We did not find sufficient data reported across the literature, press releases, or company documents for the remaining six operational projects from the GCCSI 2020 database,^2^ and these were excluded from our analysis.

### 2.2 Measures of Storage Performance

We compile estimates of four performance measures for each project. The capture rate capacity is taken as a benchmark from the reporting of the GCCSI. The capture rate is an estimate of the CO_2_ captured. Two storage rates are estimated that we label hybrid and average, due to the non-uniformity in data reporting. These are each described in Table 1 and in more detail in the Supporting Information. The year for which we found the most reporting is 2019, and we provide aggregate capacity and storage estimates for this year. We also compile time series for each project and in aggregate.

**Table 1:** Summary of Definitions for Performance Metrics

performance metric	definitions
capture rate capacity	definitions vary among projects and include the following
	(1) maximum CO_2_ captured in a particular year
	(2) maximum amount of CO_2_ that can be captured in a year based on the facility design
	(3) average capture rate for a given period
	(4) intended capture target
capture rate	an estimate of the annual amount of CO_2_ that has been captured after the project commenced
storage rate-hybrid	an estimate that uses the annual storage rate where possible (only some projects provided this data) and the average storage rate when only cumulative volumes are reported
storage rate-average	an estimated average over the lifetime of a project

### 2.3 Data Sources and Source Categorization

We compile our database using multiple sources for projects when possible. We placed these sources into three categories (Table 2), broadly corresponding to the degree of legal liability or auditing associated with the reporting. The highest degree of assurance is for category 1 data, and the lowest degree of assurance is for category 3.

**Table 2:** Summary of the Three Categories of Sources of Reporting on CO_2_ Storage with Varying Degrees of Data Assurance and Quality Control Associated with Each Category^a^

category 1	category 2	category 3
UNFCCC	corporate sustainability report	press releases
U.S. EPA	corporate ESG report	Web pages
	nongovernmental organization-prepared reports	company presentations

a Category 1 sources have the highest degree of assurance, followed by categories 2 and 3.

Data in the first category are reported under authoritative legal frameworks, including the National Inventory Report submitted to the United Nations Framework Convention on Climate Change and the Greenhouse Gas Reporting Program at the U.S. Environment Protection Agency (EPA; category 1).^10,11^ These reporting frameworks follow the requirements of the institutions for quality assurance such as internal technical reviews by an expert review team and verification protocols.^12−14^ As a result, these types of international and national frameworks employ relatively rigorous quality control and assurance of the reported CO_2_ capture and storage data.

We obtain category 2 data from annual corporate sustainability or environmental, social, and governance reports that describe the quantitative performance of CCS projects. These reports are also accompanied by statements that offer some assurance, provided by an independent assurance service, e.g., KPMG. In this category, we also include the China Annual Report 2019 prepared by the Chinese Academy of Environmental Planning, an organization founded by the Chinese government.^15^

In category 3 sources, we include company Web sites, press releases, and presentations that provide information about capture and storage rates, but without an associated statement of legal assurance or quality control of the data. The categories are summarized in Table 2.

### 2.4 Data Analysis

As described above, we report data in four categories: capture rate capacity, capture rate, storage rate-hybrid, and storage rate-average. These are estimates based on data that can be gathered from publicly available resources provided by operators. The exclusion of projects that have not publicly reported data may result in these estimates being smaller than the quantity of CO_2_ stored in practice. We provide these values in units of megatonnes of CO_2_ per year and report the capture and storage rates as a fraction of the capture rate capacity. We also quantify the fraction of the capture rate that is sequestered. Finally, we calculate the average annual growth rate in capture rate capacities and storage rates between 1996 and 2020 using the aggregate capture rate capacity time series and the aggregate storage hybrid time series.

For each project, we compile data from multiple sources with varying levels of assurance. As a result, several projects in our database have data collected for each performance metric found using more than one category of source. We record all collected data and indicate their respective source category. Data associated with the most rigorously assured source for each project are used to calculate the measures used in comparing between projects. We provide a measure of uncertainty by recalculating the aggregate using data associated with sources that have the lowest level of assurance. In this approach, uncertainty is a reflection of the deviation that exists in the reporting among various sources. Different sources often report the same numbers. As a result, performance metrics for each project have no more than two entries of data. Therefore, we do not report means or standard deviations because they are likely statistically irrelevant.

## 3 Results and Discussion

### 3.1 Aggregate Rates and Cumulative Storage

**Figure 1:**
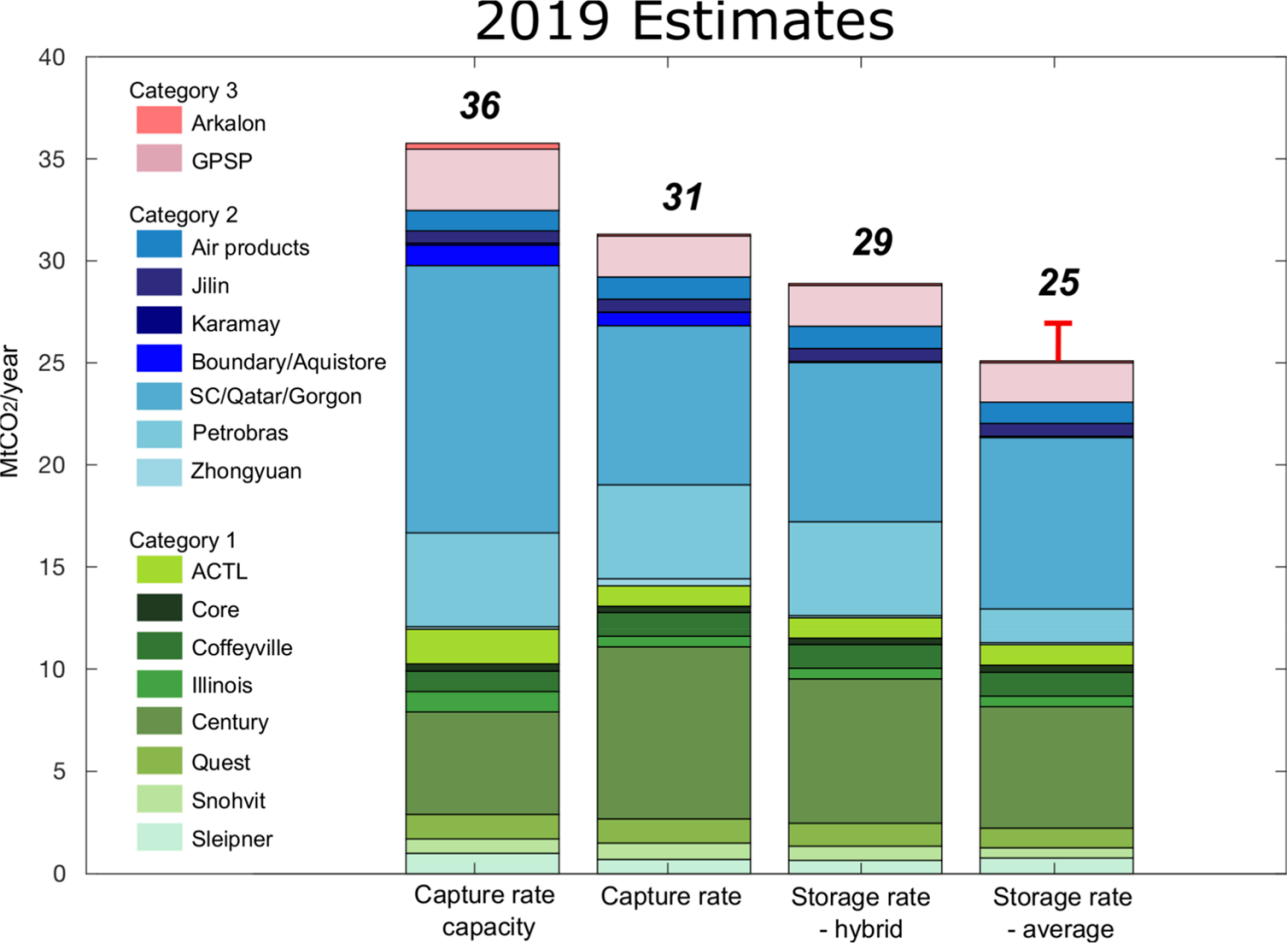
Plot comparing the compiled 2019 estimates of capture rate capacity, capture rate, average storage rate, and storage rate for 20 operational CCS projects. The range of colors illustrates the distribution of projects across the three reporting categories (definitions of each category are summarized in Table 1) and shows the maximum reporting category identified for each project. The uncertainty bar (red) can be illustrated for only the storage rate-average. Definitions of rates compared here and source categorization are provided in Materials and Methods. Summary statistics are provided in Tables 1 and 2 of the Supporting Information.

Here, we show comparisons among the 2019 aggregate capture rate capacity, capture rate, storage rate-hybrid, and storage rate-average for the 20 CCS projects for which we found information (Figure 1; full data are provided in the Supporting Information). The total capture rate capacity in 2019 is 36 Mt of CO_2_ year^–1^. Including all categories (1–3) of data for these projects, we estimate an aggregate capture rate of 31 Mt of CO_2_ year^–1^, 88% of the aggregate capture rate capacity. The aggregate storage rate-hybrid is 29 Mt of CO_2_ year^–1^ (81% of the aggregate capture rate capacity and 92% of the aggregate capture rate). The aggregate storage rate-average is 25 Mt of CO_2_ year^–1^, representing 70% of the aggregate capture rate capacity or 80% of the aggregate capture rate. Notably, we find that data for >90% of the estimated capture and storage rates fall into category 1 or 2 sources (shades of green and blue in Figure 1).

Variation in reported values among sources is reported and shown as an uncertainty bar over the average storage rate estimate in Figure 1. For the storage rate-hybrid, variations in estimates using different categories of sources are entirely due to the significant figures reported by different sources. For the storage rate-average, the variation is more significant when considering the varying sources, particularly for the Century project. This is mostly due to the high annual storage data reported by the operator Occidental Petroleum of 12.4 Mt of CO_2_ year^–1^ in 2017 (category 2 source)^16^ compared to the data reported in the EPA database (Table 2 of the Supporting Information).^17,18^ Thus, for the most part, there is consistency in reporting when multiple channels of reporting have been used.

### 3.2 Annual Reported Storage Rates for the Period of 1996–2020

**Figure 2:**
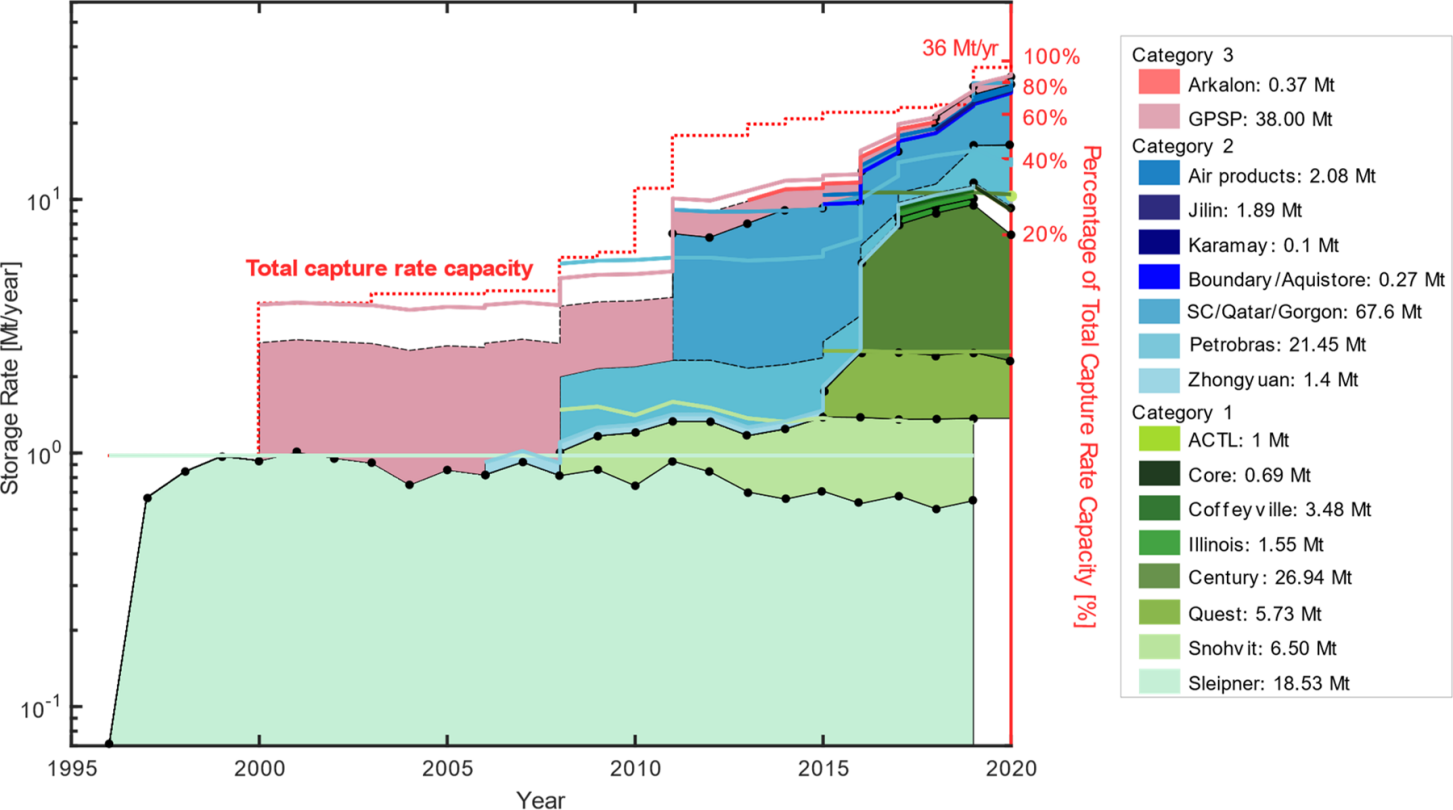
Stacked times series of annual CO_2_ storage between 1996 and 2020 to show the overall trend in storage operations. The annual storage rate (black smooth lines joined by dots) is compared with the capture rate capacity (colored lines) for Sleipner, Snohvit, Quest, Century, and combined Shute Creek, Qatar, and Gorgon. A black dashed line illustrates time series compiled using the average storage rate as no specified annual storage was reported for these projects. The annual total capture rate capacity is indicated by the red dotted line that culminates in a value of 36 Mt of CO_2_ year^–1^ in 2020. Note that the GCCSI indicates that the Shute Creek facility began operation in 1986 with a stated capture capacity of 7 Mt of CO_2_ year^–1^. However, we found storage data for Shute creek starting in only 2011, and this is when it is included in the total capture capacity time series. Similarly, the GCCSI indicates capture capacity for Petrobras starting in 2013; however, we have found storage data since 2008, and this is where that time series begins contributing to the total capture capacity. The area under each time series represents the cumulative stored, and the value is provided in the legend. The three ranges of colors are associated with the maximum source category identified for each project, and the definition of each category corresponds to the summary provided in Table 1. The green dot represents the storage rate for the Alberta Carbon Trunk Line projects, including Nutrien and Sturgeon, which began operation only in 2020. Note that the vertical axis is only using the logarithmic scale so that all the projects can be seen in the graph. The bars in Figure 1 provide a better visual of the relative project size. Individual times series of projects are available in the Supporting Information.

Seventeen time series of projects for the time period of 1996–2020 are compiled in Figure 2. We illustrate differences between times series of specified annual storage data for some projects (black line joined with dots in Figure 2) and their associated capture rate capacities (colored lines in Figure 2). Our results show that 12 of 20 projects report storage rates (average or annual storage) that are <85% of their capture rate capacity in 2019. These are Sleipner, Century, Illinois, ACTL projects, Zhongyuan, combined estimates of Shute Creek, Gorgon, and Qatar, Karamay, Great Plains Synfuel Plant (GPSP), Arkalon, and Aquistore. Taking the second year of operation at Sleipner (i.e., 1998) as our initial point (to avoid the initial ramp up in operation at Sleipner that would skew the average growth rate), we find the average annual growth for aggregate capture rate capacity has been 24.6% and the annual growth in storage rates has been 23.1% using the aggregate hybrid time series.

A variety of reasons are driving these differences. For Sleipner with a declining storage rate and Snohvit with an increasing storage rate, the performance of the CCS system is linked to the production of natural gas that is the source of CO_2_. Data provided by the Norwegian Petroleum Directorate suggest Sleipner’s annual production of gas between 2000 and 2020 has been declining at an annual average rate of 14% while the annual production of Snohvit is increasing at 8%.^19,20^ Technical difficulties are a factor for some projects. The Gorgon project in Western Australia experienced a delay in start-up due to corrosion of injection pipes and problems with their water production pressure management wells. Injection rates were limited by governmental regulators.^21,22^ At the Boundary Dam capture facility, suspensions of the CCS facility occurred due to scheduled maintenance, outages at the power station, and technical difficulties with the CO_2_ compressor.^23^ For Quest, the main contributors to the reduced capture rate in 2019 were minor technical issues in the capture unit resulting in trips, planned maintenance, and periods of decreased hydrogen production demand.^24,25^ Finally, projects that have just begun operation, i.e., Qatar LNG and ACTL, may be undergoing a period of ramp-up.

There are inconsistencies in the definitions of capture rate capacity used in the reporting. Thus, the differences between capture rate capacity and observed storage amounts may not reflect the operating performance of the CCS system. At Sleipner, the capture rate capacity (1 Mt of CO_2_ year^–1^) appears to be the maximum CO_2_ captured in 2001. The discrepancy between the amount stored and the capture capacity inevitably increases over time as natural gas production declines even if the project is operating without issue. In contrast, with Snohvit, Petrobras, and Air products, the capture rate capacity (0.7, 4.6, and 1 Mt of CO_2_ year^–1^, respectively) appears to be reported as an intended target and does not reflect the technical capture capacity of the system. As a result, the actual capture and storage rates can at times exceed their capture capacity. For Quest, the definition is unclear. According to the most recent performance review,^25^ the percentage of CO_2_ captured from the raw hydrogen gas stream did not reach the anticipated target of 80%. It is unclear whether this is equivalent to the reported capture capacity of 1.2 Mt of CO_2_ year^–1^. At Century, Illinois, Shute Creek, Gorgon, and Qatar, the capture rate capacity appears to be the maximum design capacity of the capture facility. For these projects, no information about the discrepancies between capture capacity and storage rates was found. Similarly, for projects that reported only a single figure of cumulative storage (Zhongyuan, Coffeyville, Aquistore, Jilin, GPSP, Karamay, and Arkalon), we could not critically evaluate the operating performance. The estimates of storage figures suggest that the use of capture capacity as a proxy for storage rates may overestimate the amount of CO_2_ stored by 19–30%. At the same time, there are no systematic trends in the metrics. The reasons for differences in these figures remain specific to each project.

The cumulative storage of CO_2_ (between 1996 and 2020) is estimated to be 197 Mt, combining all reporting categories (colored area in Figure 2); this is significant, equivalent to what had been achieved by solar photovoltaics by 2015.^26,27^ The estimate storage rate-hybrid of 29 Mt of CO_2_ year^–1^ is approximately half of the estimated emissions avoided as a result of deployment of solar photovoltaics in the United States in 2018.^29^ The annual growth in CCS deployment required to achieve gigatonne-scale impacts by 2050 is similar to current rates of growth in solar photovoltaics.^28^ The large-scale nature of each CCS installation has been identified as a significant barrier to growth.^30^ However, the benefit of large projects is observed here in the disproportionately large climate impact of a technology early in its development, with only scores of operational projects.

### 3.3 Implications

Our database provides further insight into the status of CCS, and it can be used as a reference in the near term for understanding the total performance of project chains. These data provide a snapshot of a climate change mitigation technology that is emerging but nonetheless already contributing significantly to emissions mitigation today. The significant difference between reported storage data and the more frequently reported capture capacity reveals an important gap in the availability and use of data necessary for evaluating the climate change impact of CCS. While the use of capture capacity as a proxy overstates the storage rate, the growth in capture capacity and the growth in storage rates track with each other. A number of studies have analyzed existing growth in the context of climate change mitigation scenarios, generally identifying that CCS deployed by midcentury in these projections will be difficult to achieve, whereas current growth is significant with very large-scale mitigation achieved by the end of the century.^31−34^

The need for consistent reporting on storage performance by industry projects is evident. The framework should include key details necessary for evaluating storage performance, including clarity in definitions of project sizes and the identification of a common nomenclature, e.g., capture capacity, identifying annual quantities of CO_2_ stored for individual projects without aggregating projects, specifying the quality control of measurements at the site level to assess uncertainty, and an association of the capture facility with one or multiple storage operators. Specific measures that would be useful in such a reporting framework include (1) the intended capture rate capacity, (2) the maximum capture rate capacity, (3) the annual capture of CO_2_, (4) the annual transport of CO_2_, (5) the annual storage of CO_2_, (6) quality assurance measures such as auditing by third parties and quantification of key uncertainties, and (7) reasons for any offline periods where the CCS facility could not operate as intended. This would enable the accurate assessment and monitoring of climate change mitigation benefits explicitly attributed to CCS operations.^30−32^

## Supporting Information Available

Additional methods, materials, and database, including a description of performance metrics, summary statistics for capture and storage comparisons, and the compiled geological database for individual projects with their associated time series (PDF)
Additional raw data of the geological CO_2_ storage database (XLSX)

## Abbreviations

CCS: carbon capture and storage
CO_2_: carbon dioxide
EOR: enhanced oil recovery
EPA: Environmental Protection Agency
GCCSI: Global Carbon Capture and Storage Institute
GHG: greenhouse gas
GPSP: Great Plains Synfuel Plant
IPCC: International Panel on Climate Change
Mtpa: megatonnes per annum
