# Supplementary material for: An Estimate of the Amount of Geological CO2 Storage over the Period of 1996–2020
Source: Environ Sci Technol Lett. 2022 Jul 19;9(8):693–8. doi: 10.1021/acs.estlett.2c00296 (PMC9366999; doi:10.1021/acs.estlett.2c00296)
Supplement: Supplementary file 1 — ez2c00296_si_001.pdf [file ez2c00296_si_001.pdf]

An estimate of the amount of geological CO_2 storage over the period 1996-2020

Yuting Zhang*
Department of Earth Science and Engineering
Imperial College London
Prince Consort Road
South Kensington
London
SW7 2BP
+44 7446137581
yuting.zhang16@imperial.ac.uk

Christopher Jackson
Department of Earth Science and Engineering
Imperial College London
c.jackson@imperial.ac.uk

Samuel Krevor
Department of Earth Science and Engineering
Imperial College London
s.krevor@imperial.ac.uk

SUPPORTING INFORMATION

The supporting information includes a detailed description of performance metrics used to compare capture and storage operations of commercial-scale CCS projections, the summary statistics for Figure 1 in Section 3.1 of main text, and the detailed geological database of individual projects alongside with its associated time-series.

Description of Performance Metrics

The capture rate capacity is obtained from the GCCSI's report for the period 2019-2020. Capture rate capacity can have a variety of meanings for different projects, including the maximum quantity of CO_2 that has been captured in a year during its operational lifetime, the maximum amount of CO_2 that can be captured in a year based on the facility design, the average capture rate for a given period, and the intended capture target for a year. Despite the varied meanings, we refer to this figure as the capture rate capacity and use it as a reference for comparison because of its widespread use as a measure of project size.

The capture rate is an estimate of the annual amount of CO_2 that has been captured after the project commenced. Of the captured amount, some may be recycled or re-used for producing chemicals. Therefore, it is necessary to additionally distinguish the amount of CO_2 that is geologically sequestered from the initial capture rate. However, for many projects, the capture rate is not reported. In this case, either the reported annual storage rate or the lifetime average from the project cumulative storage is used as the capture rate for the project.

Due to a lack of uniformity in the data reported we use two metrics to compare the storage performance. The storage rate – average is an estimated average over the lifetime of a project. This was calculated using either the reported cumulative storage or the sum of annual storage reported for projects. The storage rate – hybrid is an estimate that uses the annual storage rate where possible (only some projects provided this data) and the average storage rate for projects that only provided the cumulative storage.

Summary Statistics

Table 1: Summary statistics for data presented in Figure 1 of main text differentiating the proportion of estimates for each performance metric that is associated with the three categories of sources. Comparison between the capture rate capacity with other key performance metrics as well as the proportions of aggregate capture rate that is translated into storage are also provided.

	2019 capture and storage rates			
Source Category	Capture rate capacity [$MtCO_2\ yr^{-1}$]	Capture rate [$MtCO_2\ yr^{-1}$]	Storage rate – hybrid [$MtCO_2\ yr^{-1}$]	Storage rate – average [$MtCO_2\ yr^{-1}$]
Category 1	11.95	14.11	12.51	11.19

Category 2	20.52	15.22	14.28	11.89
Category 3	3.29	2.09	2.09	2.02
**Total**	**35.76**	**31.42**	**28.89**	**25.09**
% of aggregate capture rate capacity		88%	81%	70%
% of aggregate capture rate			92%	80%

Table 2: Summary statistics for four projects that have multiple categories of sources collected for various performance metrics. The upper and lower bound of aggregate estimates for each performance metric are also indicated. Uncertainty is estimated relative to a baseline which is provided by the reporting with the highest degree of assurance, e.g., category 1 data for a project will provide the baseline, variation from that baseline is calculated for category 2 and 3 data. The storage rate - average that are indicated in bold are obtained from the reported cumulative storage reported as opposed to the sum of year-on-year data. N/A indicate where no meaningful comparison can be derived from different estimates of cumulative storage because the number of years included in the averaging period is not consistent.

	2019 Storage rates, cumulative storage, and reporting variation							
CO_2 capture facility	Storage rate– hybrid [MtCO_2 yr^{-1}]		Storage rate – average [MtCO_2 yr^{-1}]		Cumulative storage [MtCO_2]		Averaging Period	Source category
Quest	1.128	Baseline	0.96	Baseline	4.8	Baseline	2015-2019	1
	1.13	+0.2%	0.9	-6.25%	5.39	+12%	2016-2020	2 & 3
Sleipner + Snhovit	0.65 + 0.7	Baseline	0.77 + 0.5	Baseline	18.5 + 6.5	N/A	1996-2019	1
	1.37	-1.5%	**1.1**	-13%	26.2		1996-2020	2
Illinois Industrial CCS	0.52	Baseline	0.52	Baseline	1.55	N/A	2017-2019	1
	0.52	0	0.52	0	1.042		2019-2020	2
Century (Denver + Hobbs)	3.39 + 3.66	Baseline	3.232 + 2.70	Baseline	16.16 + 10.78	N/A	2016-2020	1
	7.1	-0.7%	**8.56**	+44%	25.66		2017-2019	2
Overall aggregate (all 20 projects)	28.89	Baseline	25.10	Baseline	196.68	Baseline		Highest assurance available
Overall aggregate (all 20 projects)	28.97	+0.28%	27.02	+7.6%	196.68	0		Lower assurance

Geological CO_2 storage Database

The database includes each individual capture facility and its associated time series of CO_2 storage operations either using the reported annual storage rate or the average storage rate for projects where only the cumulative storage is provided (Table 3-16). We show comparisons between the storage operation with the stated capture rate for the year 2019 (Figure 1 -14). The aggregate total for each estimate that we evaluate: the capture rate capacity, capture rate, storage rate– hybrid, storage rate – average over project lifetime, and cumulative storage is also provided in Table 17.

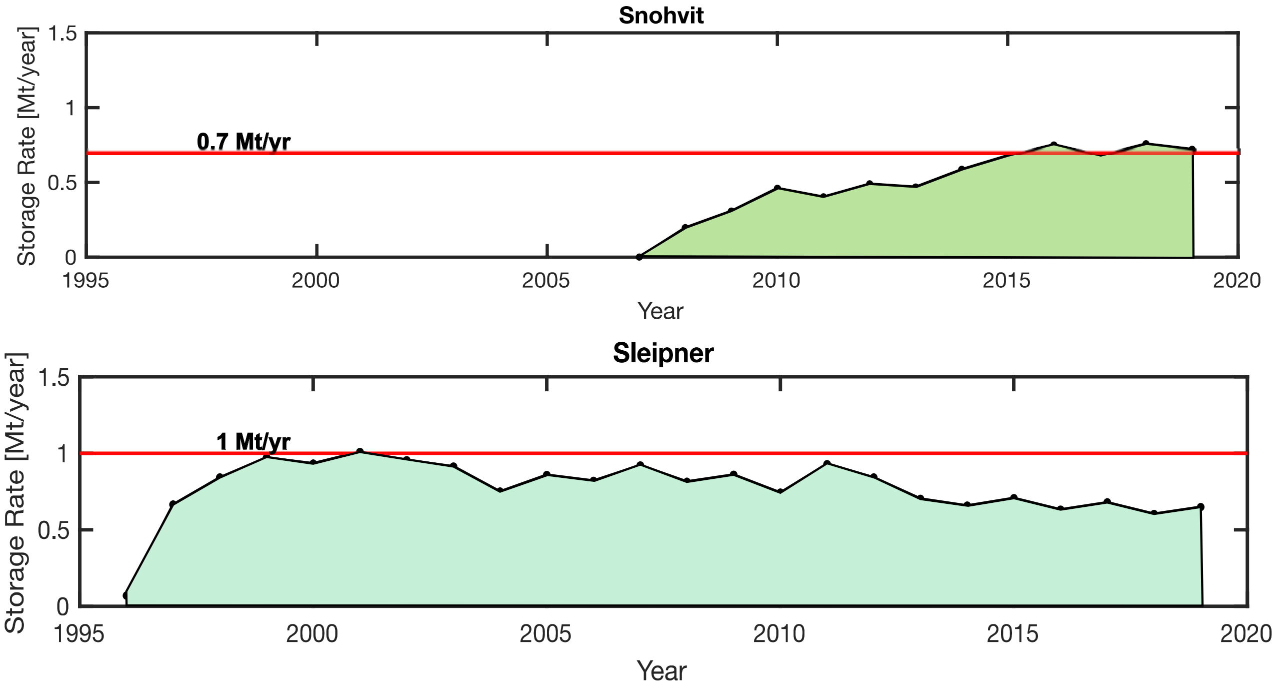

Figure 1: Times series of CO_2 storage between 1996 – 2020 to show the overall trend in annual storage operations for Sleipner and Snohvit (black smooth lines joined by dots) and the comparison with stated capture rate capacities (red line) is for 2019. The area under each time represents the cumulative storage. The colours are associated with the maximum source category identified for each project and the definition of each category corresponds to the summary provided in Table 1 in the main text. Summary statistics are provided in Table 3 of Supporting Information.

Table 3: The capture rate capacity stated for 2019-2020 is sourced from the GCCSI global status of CCS 2020" report (GCCSI, 2020). The capture rate estimated here is determined based on 1) individual sources (indicated with an asterisk), or 2) the storage rate - hybrid, depending on the availability of data. Multiple sources and data for each project are separated by thin dashed lines. The storage rate - average is calculated based on the reported cumulative storage over the number of years specified in the Period column. We indicate the categories for each source

with source categories defined in Table 1 in the main text. The storage rate– hybrid uses annual storage reported for 2019 where possible and average storage rate for projects that only report cumulative storage. The colour in the Sources column corresponds to the colour introduced in Table 1 in the main text and indicates the maximum category of sources collected for each capture project. Where there are multiple sources available for each project, data that are highlighted in red (associated with a lower level of assurance) are used to calculate uncertainty but are not included in the final aggregate estimate used for comparison in Table 17 or in figure 1 of Supporting Information.

Country	Storage type	CO_2 Capture Facility	Capture rate Capacity 2019-2020 [Mt yr^{-1}]	Capture Rate (* when reported, else from storage rates) [Mt yr^{-1}]	Associated CO_2 storage facility/operator	Storage Rate - hybrid (* when annual storage is reported, else from storage rate in 2019 - average) [Mt yr^{-1}]	Storage Rate - average over project lifetime [Mt yr^{-1}]	Cumulative storage [Mt]	Period	Source Categorisation	Sources	Notes
		Sleipner	1	0.7*		0.65*	0.77	18.5	1996-2019	1	50	
		Snohvit	0.7	0.8*		0.7*	0.5	6.5	2007-2019	1	50	
Norway	Geological Storage	Sleipner + Snohvit	1.7	1.5	Equinor	1.37*	1.1	26.2	1996-2020	2	51	Equinor annual report provided the aggregate annual data for Sleipner and Snohvit without differentiation

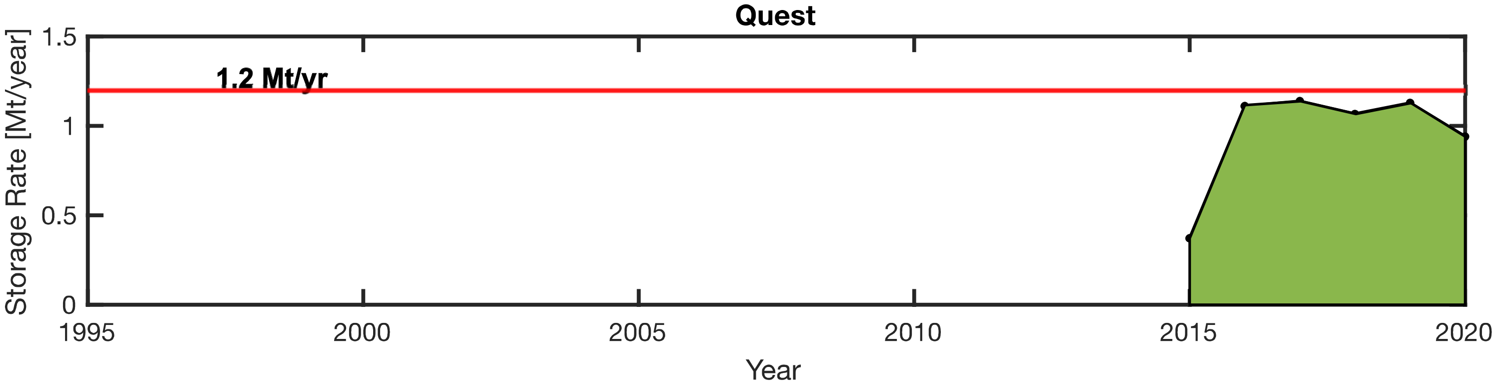

Figure 2: Times series of CO_2 storage between 2015 – 2020 to show the overall trend in annual storage operations for Quest (black smooth lines joined by dots) and the comparison with stated capture rate capacities (red line) is for 2019. The area under the time series represents the cumulative storage. The colours are associated with the maximum source category identified for each project and the definition of each category corresponds to the summary provided in Table 1 in the main text. Summary statistics are provided in Table 4 of Supporting Information.

Table 4: The capture rate capacity stated for 2019-2020 is sourced from the GCCSI global status of CCS 2020" report (GCCSI, 2020). The capture rate estimated here is determined based on 1) individual sources (indicated with an asterisk), or 2) the storage rate - hybrid, depending on the availability of data. Multiple sources and data for each project are separated by thin dashed lines. The storage rate - average is calculated based on the reported cumulative storage over the number of years specified in the Period column. We indicate the categories for each source with source categories defined in Table 1 in the main text. The storage rate– hybrid uses annual storage reported for 2019 where possible and average storage rate for projects that only report cumulative storage. The colour in the Sources column corresponds to the colour introduced in Table 1 in the main text and indicates the maximum category of sources collected for each capture project. Where there are multiple sources available for each project, data that are highlighted in red (associated with a lower level of assurance) are used to calculate uncertainty but are not included in the final aggregate estimate used for comparison in Table 17 of Supporting Information. The annual storage for 2019-2020 – 0.94 MtCO2 yr^{-1} reported by Shell Sustainability Report [39] is however included in Figure 2 of Supporting Information.

Country	Storage type	CO_2 Capture Facility	Capture rate Capacity 2019-2020 [Mt yr^{-1}]	Capture Rate (* when reported, else from storage rates) [Mt yr^{-1}]	Associated CO_2 storage facility/operator	Storage Rate - hybrid (* when annual storage is reported, else from storage rate in 2019 - average) [Mt yr^{-1}]	Storage Rate - average over project lifetime [Mt yr^{-1}]	Cumulative storage [Mt]	Period	Source Categorisation	Sources
Canada	geological storage	Quest	1.2	1.182*	Quest Shell	1.13	0.9	5.39	2016-2020	2	45
										3	46

						1.128*	0.96	4.8	2015-2019	1	24

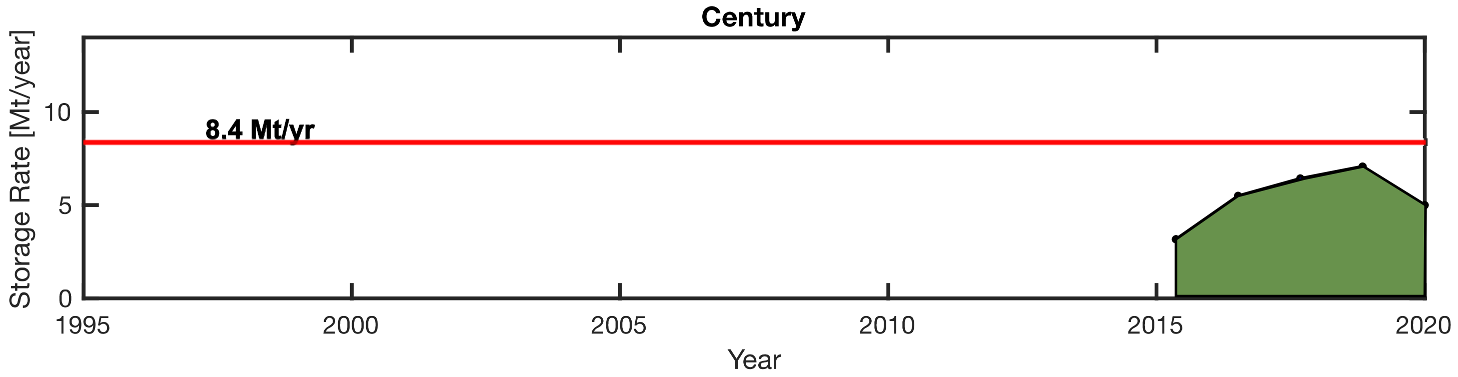

Figure 3: Times series of CO_2 storage between 2016 – 2020 to show the overall trend in annual storage operations for Quest (black smooth lines joined by dots) and the comparison with stated capture rate capacities (red line) is for 2019. The area under the time series represents the cumulative storage. The colours are associated with the maximum source category identified for each project and the definition of each category corresponds to the summary provided in Table 1 in the main text. Summary statistics are provided in Table 5 of Supporting Information.

Table 5: The capture rate capacity stated for 2019-2020 is sourced from the GCCSI global status of CCS 2020" report (GCCSI, 2020). The capture rate estimated here is determined based on 1) individual sources (indicated with an asterisk), or 2) the storage rate - hybrid, depending on the availability of data. Multiple sources and data for each project are separated by thin dashed lines. The storage rate– average is calculated based on the reported cumulative storage over the number of years specified in the Period column. We indicate the categories for each source with source categories defined in Table 1 in the main text. The storage rate – hybrid uses annual storage reported for 2019 where possible and average storage rate for projects that only report cumulative storage. The colour in the Sources column corresponds to the colour introduced in Table 1 in the main text and indicates the maximum category of sources collected for each capture project. Where there are multiple sources available for each project, data that are highlighted in red (associated with a lower level of assurance) are used to calculate uncertainty but are not included in the final aggregate estimate used for comparison in Table 17 or in Figure 3 of Supporting Information.

Country	Storage type	CO_2 Capture Facility	Capture rate Capacity 2019-2020 [Mt yr^{-1}]	Capture Rate (* when reported, else from storage rates) [Mt yr^{-1}]	Associated CO_2 storage facility/operator	Storage Rate - hybrid (* when annual storage is reported, else from storage rate in 2019 - average) [Mt yr^{-1}]	Storage Rate - average over project lifetime [Mt yr^{-1}]	Cumulative storage [Mt]	Period	Source Categorisation	Sources	Notes
US	EOR	Century	5	8.4*	Occidental Petroleum	7.1*	8.55	25.66	2017-2019	2	16	Occidental Petroleum Sustainability report

					Denver Unit	3.39*	3.232	16.16	2016-2020	1	17	provides the aggregate data for CO_2 storage while US EPA provides the differentiated storage data at the two unit sites that are operated by Occidental Petroleum
					Hobbs Unit	3.66*	2.695	10.78	2017-2020	1	18	

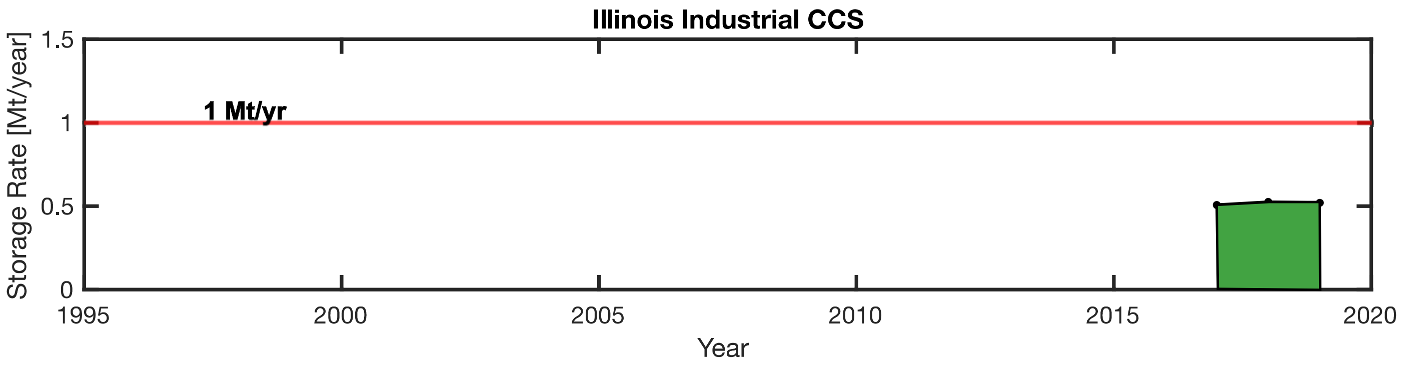

Figure 4: Times series of CO_2 storage between 2017 – 2019 to show the overall trend in annual storage operations for Illinois Industrial CCS (black smooth lines joined by dots) and the comparison with stated capture rate capacities (red line) is for 2019. The area under the time series represents the cumulative storage. The colours are associated with the maximum source category identified for each project and the definition of each category corresponds to the summary provided in Table 1 in the main text. Summary statistics are provided in Table 6 of Supporting Information.

Table 6: The capture rate capacity stated for 2019-2020 is sourced from the GCCSI global status of CCS 2020" report (GCCSI, 2020). The capture rate estimated here is determined based on 1) individual sources (indicated with an asterisk), or 2) the storage rate - hybrid, depending on the availability of data. Multiple sources and data for each project are separated by thin dashed lines. The storage rate– average is calculated based on the reported cumulative storage over the number of years specified in the Period column. We indicate the categories for each source with source categories defined in Table 1 in the main text. The storage rate – hybrid uses annual storage reported for 2019 where possible and average storage rate for projects that only report cumulative storage. The colour in the Sources column corresponds to the colour introduced in Table 1 in the main text and indicates the maximum category of sources collected for each capture project. Where there are multiple sources available for each project, data that are highlighted in red (associated with a lower level of assurance) are used to calculate uncertainty but are not included in the final aggregate estimate used for comparison in Table 7 or in Figure 4 of Supporting Information.

Country		CO_2 Capture Facility	Capture rate Capacity 2019-2020 [Mt yr^{-1}]	Capture Rate (* when reported, else from storage	Associated CO_2 storage facility/operator	Storage Rate - hybrid (* when annual storage is reported, else from storage rate in 2019 - average) [Mt yr^{-1}]	Storage Rate - average over project lifetime [Mt yr^{-1}]	Cumulative storage [Mt]	Period	Source Categorisation	Sources

	Storage type			rates) [Mt yr^{-1}]							
US	Dedicated geological storage	Illinois Industrial CCS	1	0.52	Illinois ADM	0.52*<br>0.522*	0.52<br>0.52	1.55<br>1.042	2017-2019<br>2019-2020	1<br>2	52<br>53

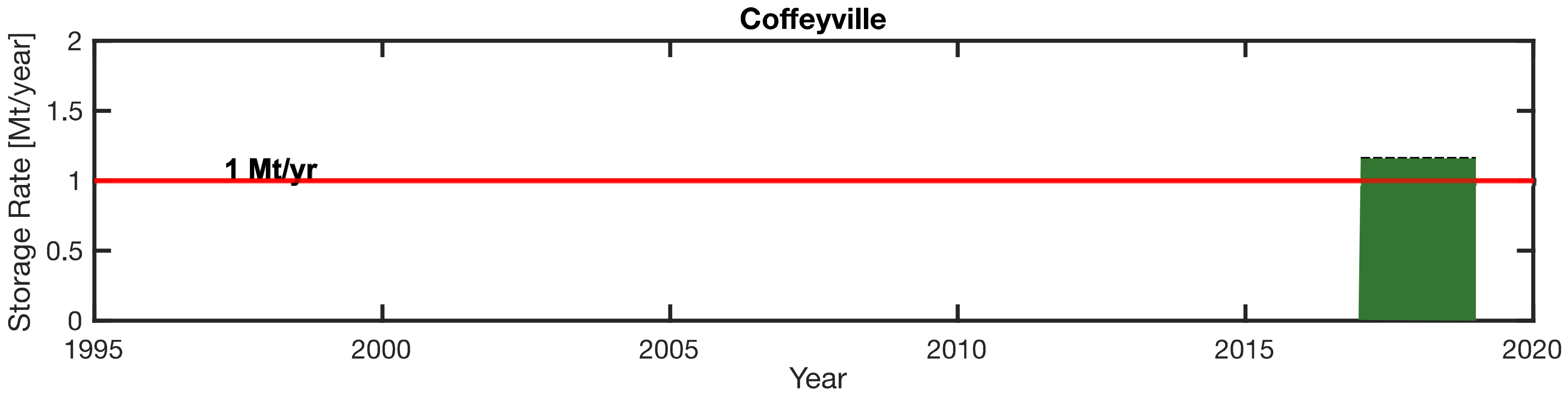

Figure 5: Times series of CO_2 storage between 2017 – 2019 to show the overall trend in annual storage operations for Coffeyville (black smooth lines joined by dots) and the comparison with stated capture rate capacities (red line) is for 2019. The area under the time series represents the cumulative storage. The colours are associated with the maximum source category identified for each project and the definition of each category corresponds to the summary provided in Table 1 in the main text. Summary statistics are provided in Table 7 of Supporting Information.

Table 7: The capture rate capacity stated for 2019-2020 is sourced from the GCCSI global status of CCS 2020" report (GCCSI, 2020). The capture rate estimated here is determined based on 1) individual sources (indicated with an asterisk), or 2) the storage rate - hybrid, depending on the availability of data. Multiple sources and data for each project are separated by thin dashed lines. The storage rate – average is calculated based on the reported cumulative storage over the number of years specified in the Period column. We indicate the categories for each source with source categories defined in Table 1 in the main text. The storage rate– hybrid uses annual storage reported for 2019 where possible and average storage rate for projects that only report cumulative storage. The colour in the Sources column corresponds to the colour introduced in Table 1 in the main text and indicates the maximum category of sources collected for each capture project.

Country		CO_2 Capture Facility	Capture rate Capacity 2019-2020 [Mt yr^{-1}]	Capture Rate (* when reported, else from storage rates) [Mt yr^{-1}]	Associated CO_2 storage facility/operator	Storage Rate - hybrid (* when annual storage is reported, else from storage rate in 2019 - average) [Mt yr^{-1}]	Storage Rate - average over project lifetime [Mt yr^{-1}]	Cumulative storage [Mt]	Period	Source Categorisation	Sources

	Storage type										
US	EOR	Coffeyville	1	1.16	North Burbank Unit	1.16	1.16	3.49	2017-2019	1	55

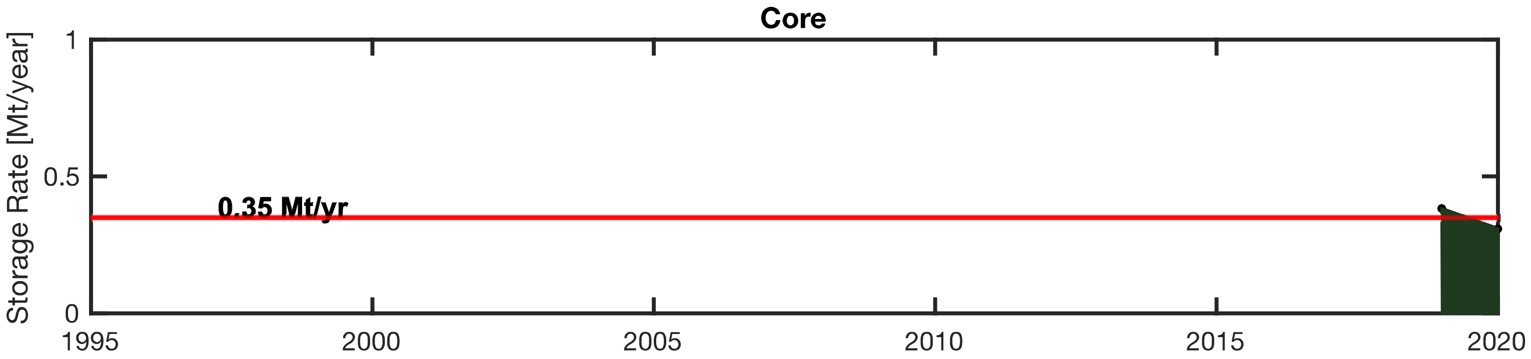

Figure 6: Times series of CO_2 storage between 2019 - 2020 to show the overall trend in annual storage operations for Core Energy (black smooth lines joined by dots) and the comparison with stated capture rate capacities (red line) is for 2019. The area under the time series represents the cumulative storage. The colours are associated with the maximum source category identified for each project and the definition of each category corresponds to the summary provided in Table 1 in the main text. Summary statistics are provided in Table 8 of Supporting Information.

Table 8: The capture rate capacity stated for 2019-2020 is sourced from the GCCSI global status of CCS 2020" report (GCCSI, 2020). The capture rate estimated here is determined based on 1) individual sources (indicated with an asterisk), or 2) the storage rate - hybrid, depending on the availability of data. Multiple sources and data for each project are separated by thin dashed lines. The storage rate– average is calculated based on the reported cumulative storage over the number of years specified in the Period column. We indicate the categories for each source with source categories defined in Table 1 in the main text. The storage rate – hybrid uses annual storage reported for 2019 where possible and average storage rate for projects that only report cumulative storage. The colour in the Sources column corresponds to the colour introduced in Table 1 in the main text and indicates the maximum category of sources collected for each capture project.

Country	Storage type	CO_2 Capture Facility	Capture rate Capacity 2019-2020 [Mt yr^{-1}]	Capture Rate (* when reported, else from storage rates) [Mt yr^{-1}]	Associated CO_2 storage facility/operator	Storage Rate - hybrid (* when annual storage is reported, else from storage rate in 2019 - average) [Mt yr^{-1}]	Storage Rate - average over project lifetime [Mt yr^{-1}]	Cumulative storage [Mt]	Period	Source Categorisation	Sources
US	EOR	Core Energy	0.35	0.35	Core Energy	0.31*	0.35	0.69	2019-2020	1	61

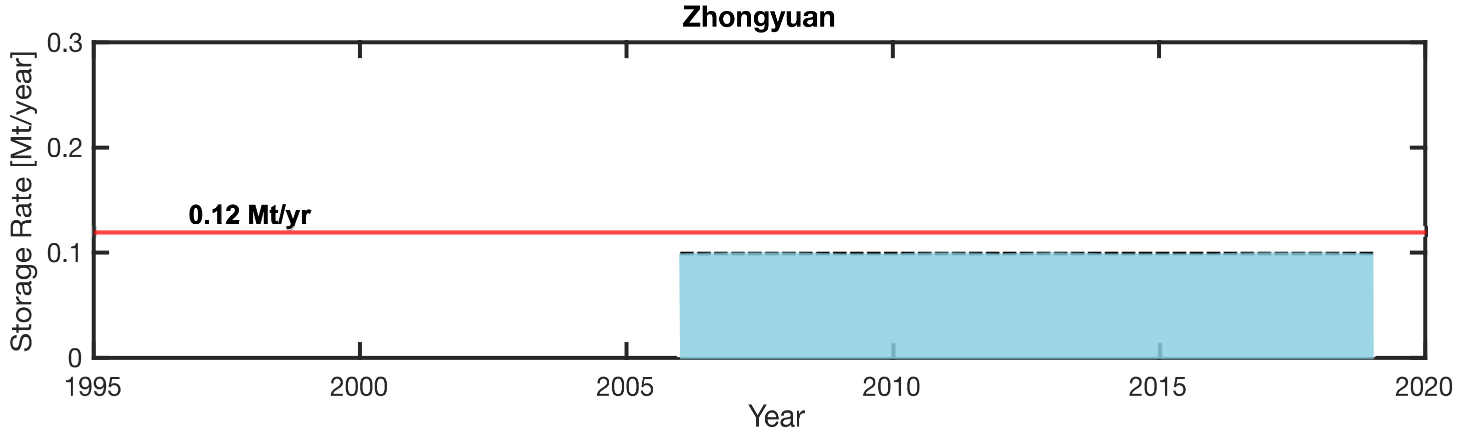

Figure 7: Times series of CO_2 storage between 2006-2019 to show the average storage operations for Zhongyuan (black dash line) and the comparison with stated capture rate capacities (red line) is for 2019. The area under the time series represents the cumulative storage. The colours are associated with the maximum source category identified for each project and the definition of each category corresponds to the summary provided in Table 1 in the main text. Summary statistics are provided in Table 9 of Supporting Information.

Table 9: The capture rate capacity stated for 2019-2020 is sourced from the GCCSI global status of CCS 2020" report (GCCSI, 2020). The capture rate estimated here is determined based on 1) individual sources (indicated with an asterisk), or 2) the storage rate - hybrid, depending on the availability of data. Multiple sources and data for each project are separated by thin dashed lines. The storage rate– average is calculated based on the reported cumulative storage over the number of years specified in the Period column. We indicate the categories for each source with source categories defined in Table 1 in the main text. The storage rate– hybrid uses annual storage reported for 2019 where possible and average storage rate for projects that only report cumulative storage. The colour in the Sources column corresponds to the colour introduced in Table 1 in the main text and indicates the maximum category of sources collected for each capture project.

Country	Storage type	CO_2 Capture Facility	Capture rate Capacity 2019-2020 [Mt yr^{-1}]	Capture Rate (* when reported, else from storage rates) [Mt yr^{-1}]	Associated CO_2 storage facility/operator	Storage Rate - hybrid (* when annual storage is reported, else from storage rate in 2019 - average) [Mt yr^{-1}]	Storage Rate - average [Mt yr^{-1}]	Cumulative storage [Mt]	Period	Source Categorisation	Sources
China	EOR	Sinopec Zhongyuan	0.12	0.35	Zhongyuan Sinopec	0.1	0.1	2.4	2006-2019	2<br>2	15<br>62

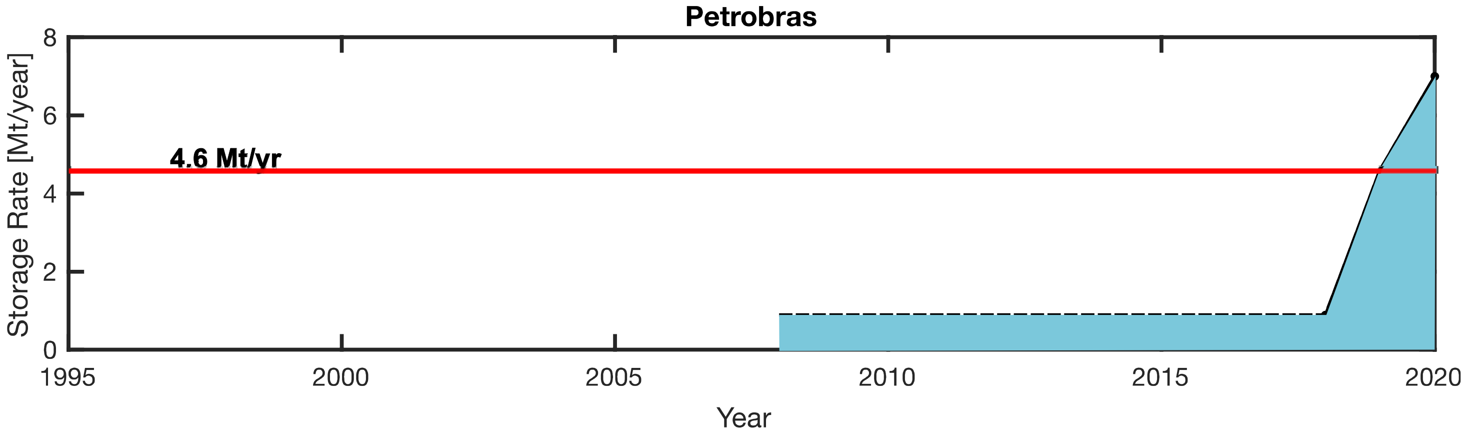

Figure 8: Times series of CO_2 storage between 2008-2018 to show the average storage operation (black dash line) and annual storage rate from 2018 -2020 (black smooth line joined by dots) for Petrobras. The comparison with stated capture rate capacities (red line) is for 2019. The area under the time series represents the cumulative storage. The colours are associated with the maximum source category identified for each project and the definition of each category corresponds to the summary provided in Table 1 in the main text. Summary statistics are provided in Table 10 of Supporting Information.

Table 10: The capture rate capacity stated for 2019-2020 is sourced from the GCCSI global status of CCS 2020" report (GCCSI, 2020). The capture rate estimated here is determined based on 1) individual sources (indicated with an asterisk), or 2) the storage rate - hybrid, depending on the availability of data. Multiple sources and data for each project are separated by thin dashed lines. The storage rate – average is calculated based on the reported cumulative storage over the number of years specified in the Period column. We indicate the categories for each source with source categories defined in Table 1 in the main text. The storage rate– hybrid uses annual storage reported for 2019 where possible and average storage rate for projects that only report cumulative storage. The colour in the Sources column corresponds to the colour introduced in Table 1 in the main text and indicates the maximum category of sources collected for each capture project.

Country	Storage type	CO_2 Capture Facility	Capture rate Capacity 2019-2020 [Mt yr^{-1}]	Capture Rate (* when reported, else from storage rates) [Mt yr^{-1}]	Associated CO_2 storage facility/operator	Storage Rate - hybrid (* when annual storage is reported, else from storage rate in 2019 - average) [Mt yr^{-1}]	Storage Rate - average over project lifetime [Mt yr^{-1}]	Cumulative storage [Mt]	Period	Source Categorisation	Sources
Brazil	EOR	Petrobras	4.6	4.6	Santos Basin Petrobtras	4.6*	1.65	21.4	2008-2020	3	35
										2	36
										3	37
										2	38

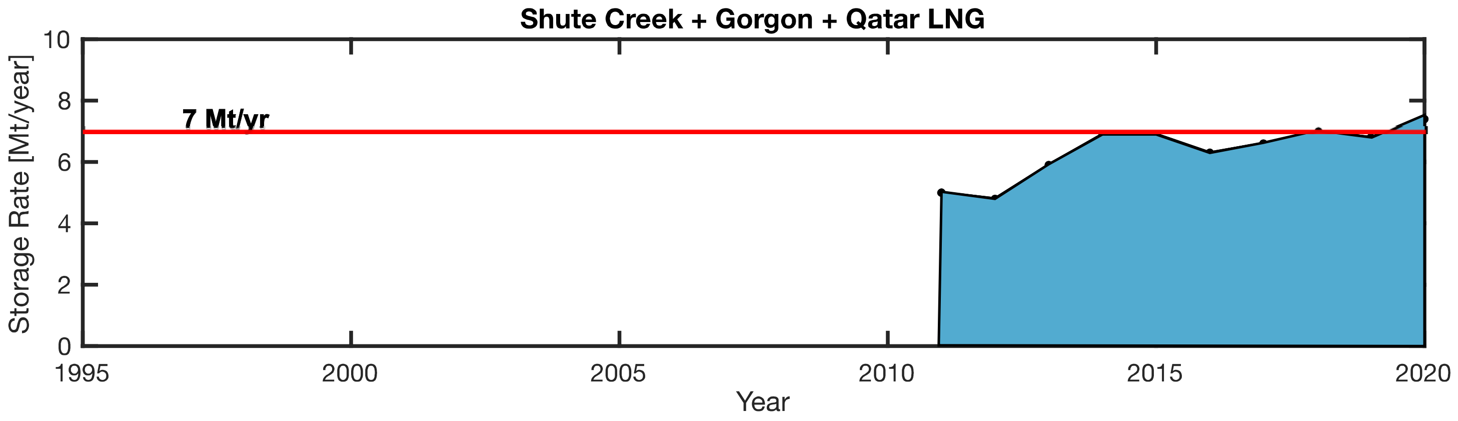

Figure 9: Times series of CO_2 storage between 2011- 2020 to show the overall trend in annual storage operations for Shute Creek, Gorgon, and Qatar LNG (black smooth lines joined by dots) and the comparison with stated capture rate capacities (red line) is for 2019. The area under the time series represents the cumulative storage. The colours are associated with the maximum source category identified for each project and the definition of each category corresponds to the summary provided in Table 1 in the main text. Summary statistics are provided in Table 11 of Supporting Information.

Table 11: The capture rate capacity stated for 2019-2020 is sourced from the GCCSI global status of CCS 2020" report (GCCSI, 2020). The capture rate estimated here is determined based on 1) individual sources (indicated with an asterisk), or 2) the storage rate - hybrid, depending on the availability of data. Multiple sources and data for each project are separated by thin dashed lines. The storage rate– average is calculated based on the reported cumulative storage over the number of years specified in the Period column. We indicate the categories for each source with source categories defined in Table 1 in the main text. The storage rate– hybrid uses annual storage reported for 2019 where possible and average storage rate for projects that only report cumulative storage. The colour in the Sources column corresponds to the colour introduced in Table 1 in the main text and indicates the maximum category of sources collected for each capture project.

Country	Storage type	CO_2 Capture Facility	Capture rate Capacity 2019-2020 [Mt yr^{-1}]	Capture Rate (* when reported, else from storage rates) [Mt yr^{-1}]	Associated CO_2 storage facility/operator	Storage Rate - hybrid (* when annual storage is reported, else from storage rate in 2019 - average) [Mt yr^{-1}]	Storage Rate - average over project life time [Mt yr^{-1}]	Cumulative storage [Mt]	Period	Source Categorisation	Sources	Notes
Qatar	Geological storage	Qatar LNG	2.1	1 (C) + 6.8 (EM)	Chevron (C) & Exxon mobile (EM)	1* (C) + 6.8* (EM)	2 (C) + 6.4 (EM)	4 (C) + 63.6 (EM)	2019-2020 (C) 2011-2020 (EM)	2	56 (EM)	Chevron only operates for the Gorgon project in Australia while Exxon mobile are involved in all three CCS projects including Shute Creek, Qatar LNG and Gorgon. However, the
Australia	Geological storage	Shute Creek	7							2	57 (C)	
US	EOR	Gorgon	4							3	58 (C)	

													Exxon Mobile sustainability report did not provide differentiated annual storage data associated with each individual project only the aggregate annual storage data in this case.

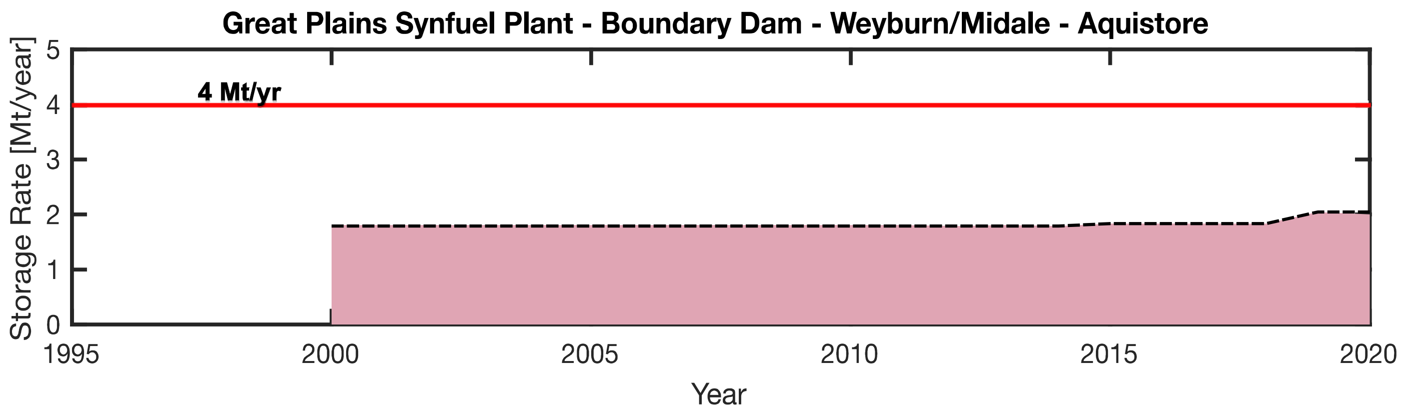

Figure 10: Times series of CO_2 storage between 2000-2020 to show the average storage operations for Aquistore/Weyburn-Midale that are associated with Great Plains Synfuel Plant/Boundary Dam capture facilities (black dash line) and the comparison with stated capture rate capacities (red line) is for 2019. The area under the time series represents the cumulative storage. The colours are associated with the maximum source category identified for each project and the definition of each category corresponds to the summary provided in Table 1 in the main text. Summary statistics are provided in Table 12 of Supporting Information.

Table 12: The capture rate capacity stated for 2019-2020 is sourced from the GCCSI global status of CCS 2020" report (GCCSI, 2020). The capture rate estimated here is determined based on 1) individual sources (indicated with an asterisk), or 2) the storage rate - hybrid, depending on the availability of data. Multiple sources and data for each project are separated by thin dashed lines. The storage rate – average is calculated based on the reported cumulative storage over the number of years specified in the Period column. We indicate the categories for each source with source categories defined in Table 1 in the main text. The storage rate – hybrid uses annual storage reported for 2019 where possible and average storage rate for projects that only report cumulative storage. The colour in the Sources column corresponds to the colour introduced in Table 1 in the main text and indicates the maximum category of sources collected for each capture project. Where there are multiple sources available for each project, data that are highlighted in red (associated with a lower level of assurance) are used to calculate uncertainty but are not included in the final aggregate estimate used for comparison in Table 17 or in Figure 10 of Supporting Information.

Country	Storage type	CO_2 Capture Facility	Capture rate Capacity 2019-2020 [Mt yr^{-1}]	Capture Rate (* when reported, else from storage rates) [Mt yr^{-1}]	Associated CO_2 storage facility/operator	Storage Rate hybrid (* when annual storage is reported, else from storage rate in 2019 - average) [Mt yr^{-1}]	Storage Rate - average over project lifetime [Mt yr^{-1}]	Cumulative storage [Mt]	Period	Source Categorisation	Sources	Notes

Canada	EOR	Boundary Dam										
US/Canada	EOR	Great Plains Synfuel Plant	1 (A) + 3 (W)	0.65*(A) +2 (W)	Project Aquistore (A) & Weyburn-Midale Whitecap Resources(W)	0.045 (A) + 2*(W)	0.045 (A) + 1.93 (W) 1.79 (W)	0.27 (A) + 5.8 (W) + 32.2 (W)	2015-2020 (A) 2018-2020 (W) 2000-2017 (W)	2<br>3<br>3<br>3<br>3<br>3	39 (Aquistore)<br>40 (Aquistore)<br>41 (Whitecap)<br>42 (Whitecap)<br>43 (Whitecap)<br>44 (Whitecap)	In this case, there are multiple capture facilities: Boundary Dam and Great Plains Synfuel Plant (GPSP) transporting captured CO_2 to the Weyburn-Midale storage site that is operated by Whitecap Resources. Additionally, a small proportion of captured CO_2 from the Boundary Dam facility is transported to the demonstration project – Aquistore for storage. However, Whitecap resources did not differentiate how much CO_2 stored was from the Boundary Dam or GPSP plant.

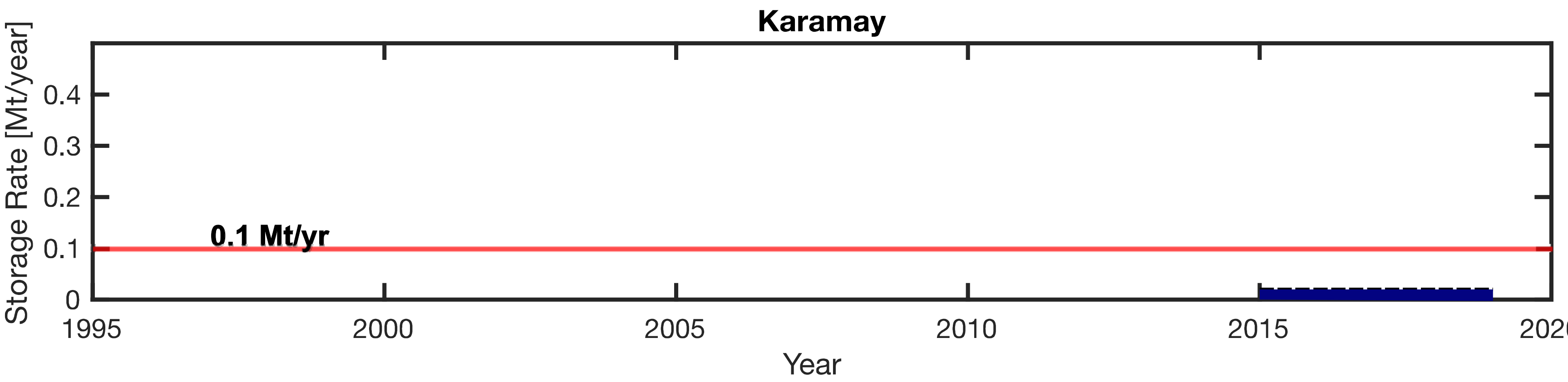

Figure 11: Times series of CO_2 storage between 2015-2019 to show the average storage operations for Karamay Dunhua (black dash line) and the comparison with stated capture rate capacities (red line) is for 2019. The area under the time series represents the cumulative storage. The colours are associated with the maximum source category identified for each project and the definition of each category corresponds to the summary provided in Table 1 in the main text. Summary statistics are provided in Table 13 of Supporting Information.

Table 13: The capture rate capacity stated for 2019-2020 is sourced from the GCCSI global status of CCS 2020" report (GCCSI, 2020). The capture rate estimated here is determined based on 1) individual sources (indicated with an asterisk), or 2) the storage rate - hybrid, depending on the availability of data. Multiple sources and data for each project are separated by thin dashed lines. The storage rate– average is calculated based on the reported cumulative storage over the number of years specified in the Period column. We indicate the categories for each source with source categories defined in Table 1 in the main text. The storage rate– hybrid uses annual storage reported for 2019 where possible and average storage rate for projects that only report

cumulative storage. The colour in the Sources column corresponds to the colour introduced in Table 1 in the main text and indicates the maximum category of sources collected for each capture project.

Country	Storage type	CO_2 Capture Facility	Capture rate Capacity 2019-2020 [Mt yr^{-1}]	Capture Rate (* when reported, else from storage rates) [Mt yr^{-1}]	Associated CO_2 storage facility/operator	Storage Rate - hybrid (* when annual storage is reported, else from storage rate in 2019 - average) [Mt yr^{-1}]	Storage Rate - average [Mt yr^{-1}]	Cumulative storage [Mt]	Period	Source Categorisation	Sources
China	EOR	Karamay Dunhua	0.1	0.1	Karamay Dunhua	0.02	0.02	0.2	2015-2019	2	15

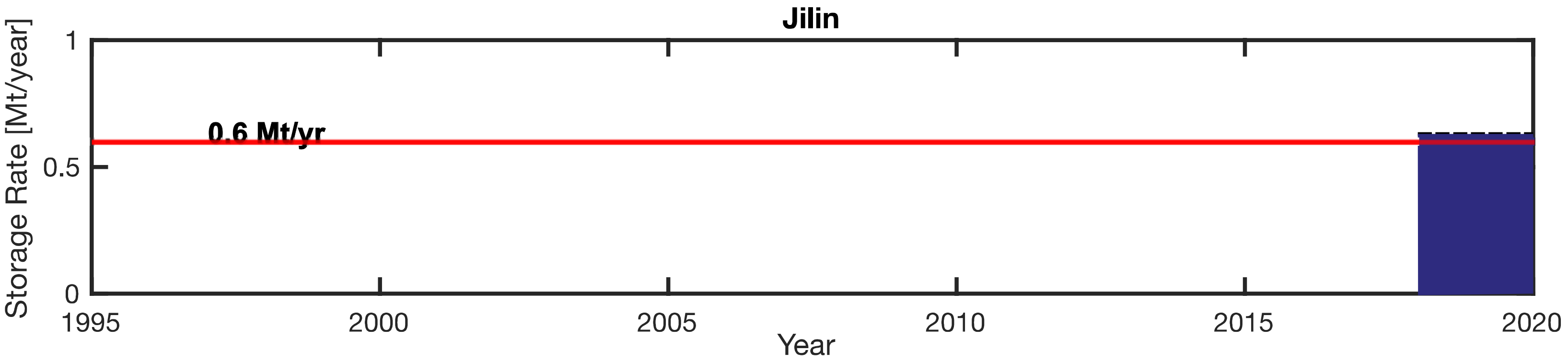

Figure 12: Times series of CO_2 storage between 2018-2020 to show the average storage operations for Karamay Dunhua (black dash line) and the comparison with stated capture rate capacities (red line) is for 2019. The area under the time series represents the cumulative storage. The colours are associated with the maximum source category identified for each project and the definition of each category corresponds to the summary provided in Table 1 in the main text. Summary statistics are provided in Table 14 of Supporting Information.

Table 14: The capture rate capacity stated for 2019-2020 is sourced from the GCCSI global status of CCS 2020" report (GCCSI, 2020). The capture rate estimated here is determined based on 1) individual sources (indicated with an asterisk), or 2) the storage rate - hybrid, depending on the availability of data. Multiple sources and data for each project are separated by thin dashed lines. The storage rate – average is calculated based on the reported cumulative storage over the number of years specified in the Period column. We indicate the categories for each source with source categories defined in Table 1 in the main text. The storage rate– hybrid uses annual storage reported for 2019 where possible and average storage rate for projects that only report cumulative storage. The colour in the Sources column corresponds to the colour introduced in Table 1 in the main text and indicates the maximum category of sources collected for each

capture project. Where there are multiple sources available for each project, data that are highlighted in red (associated with a lower level of assurance) are used to calculate uncertainty but are not included in the final aggregate estimate used for comparison in Table 17 or in Figure 12 of Supporting Information.

Country	Storage type	CO_2 Capture Facility	Capture rate Capacity 2019-2020 [Mt yr^{-1}]	Capture Rate (* when reported, else from storage rates) [Mt yr^{-1}]	Associated CO_2 storage facility/operator	Storage Rate - hybrid (* when annual storage is reported, else from storage rate in 2019 - average) [Mt yr^{-1}]	Storage Rate - average [Mt yr^{-1}]	Cumulative storage [Mt]	Period	Source Categorisation	Sources
	EOR						0.3			2	15
China		CNPC Jilin	0.6	0.63	Jilin CNPC	0.63	0.63	1.9	2018-2020	2	49

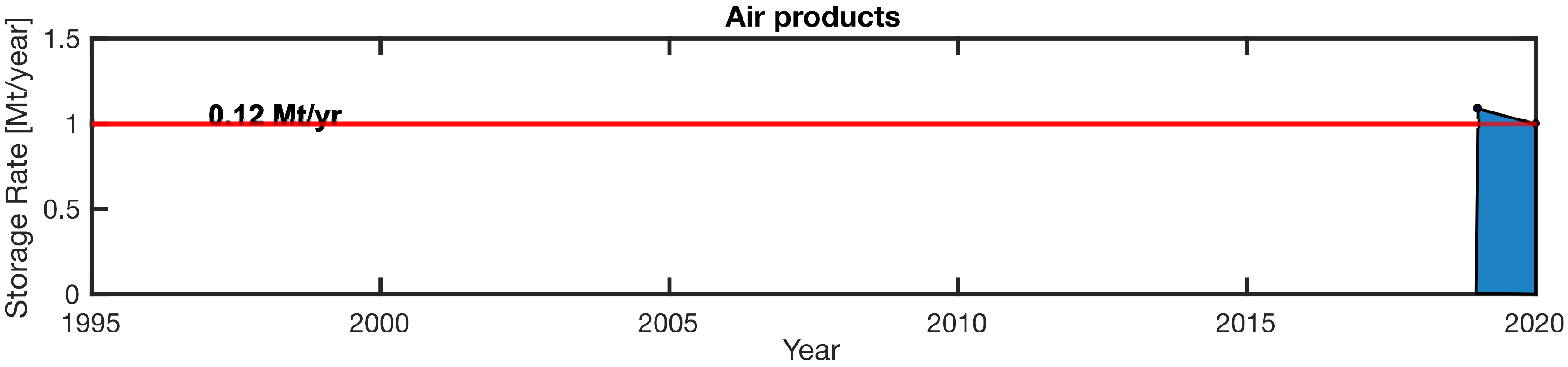

Figure 13: Times series of CO_2 storage between 2018-2020 to show the overall trend in annual storage operations for Air products (black smooth lines joined by dots) and the comparison with stated capture rate capacities (red line) is for 2019. The area under the time series represents the cumulative storage. The colours are associated with the maximum source category identified for each project and the definition of each category corresponds to the summary provided in Table 1 in the main text. Summary statistics are provided in Table 15 of Supporting Information.

Table 15: The capture rate capacity stated for 2019-2020 is sourced from the GCCSI global status of CCS 2020" report (GCCSI, 2020). The capture rate estimated here is determined based on 1) individual sources (indicated with an asterisk), or 2) the storage rate - hybrid, depending on the availability of data. Multiple sources and data for each project are separated by thin dashed lines. The storage rate– average is calculated based on the reported cumulative storage over the number of years specified in the Period column. We indicate the categories for each source with source categories defined in Table 1 in the main text. The storage rate– hybrid uses annual storage reported for 2019 where possible and average storage rate for projects that only report cumulative storage. The colour in the Sources column corresponds to the colour introduced in Table 1 in the main text and indicates the maximum category of sources collected for each capture project.

Country		CO_2 Capture Facility	Capture rate Capacity	Capture Rate (* when reported, else	Associated CO_2 storage facility/operator	Storage Rate - hybrid (* when annual storage is reported, else	Storage Rate- average over	Cumulative storage [Mt]	Period	Source Categorisation	Sources

	Storage type		2019-2020 [Mt yr^{-1}]	from storage rates) [Mt yr^{-1}]		from storage rate in 2019 - average) [Mt yr^{-1}]	project lifetime[Mt yr^{-1}]				
US	EOR	Air products	1	1.09	Gulf Coast Denbury	1.09*	1.04	2.08	2019-2020	2<br>3	59<br>60

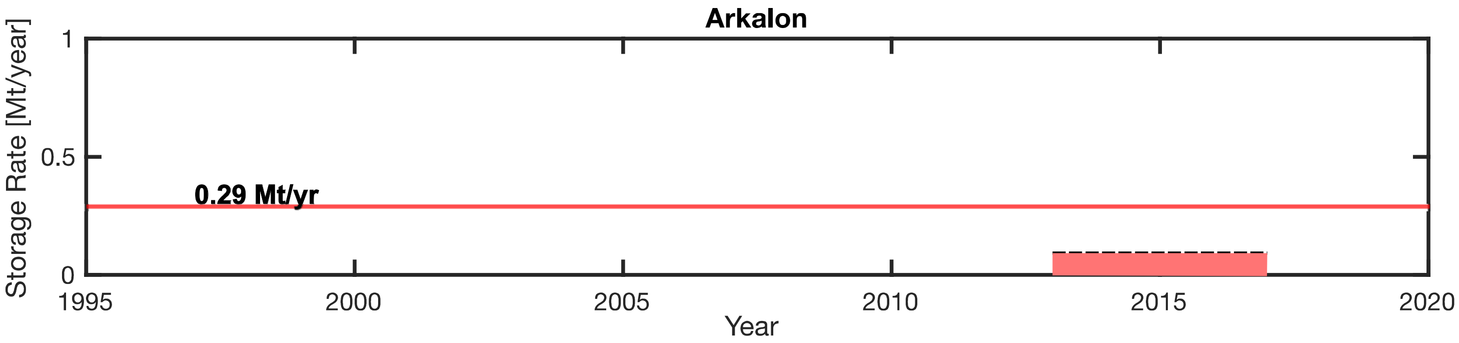

Figure 14: Times series of CO_2 storage between 2013-2017 to show the average storage operations for Arkalon (black dash line) and the comparison with stated capture rate capacities (red line) is for 2019. The area under the time series represents the cumulative storage. The colours are associated with the maximum source category identified for each project and the definition of each category corresponds to the summary provided in Table 1 in the main text. Summary statistics are provided in Table 16 of Supporting Information.

Table 16: The capture rate capacity stated for 2019-2020 is sourced from the GCCSI global status of CCS 2020" report (GCCSI, 2020). The capture rate estimated here is determined based on 1) individual sources (indicated with an asterisk), or 2) the storage rate - hybrid, depending on the availability of data. Multiple sources and data for each project are separated by thin dashed lines. The storage rate– average is calculated based on the reported cumulative storage over the number of years specified in the Period column. We indicate the categories for each source with source categories defined in Table 1 in the main text. The storage rate– hybrid uses annual storage reported for 2019 where possible and average storage rate for projects that only report cumulative storage. The colour in the Sources column corresponds to the colour introduced in Table 1 in the main text and indicates the maximum category of sources collected for each capture project.

Country	Storage type	CO_2 Capture Facility	Capture rate Capacity 2019-2020 [Mt yr^{-1}]	Capture Rate (* when reported, else from storage rates) [Mt yr^{-1}]	Associated CO_2 storage facility/operator	Storage Rate - hybrid (* when annual storage is reported, else from storage rate in 2019 - average) [Mt yr^{-1}]	Storage Rate - average over project lifetime [Mt yr^{-1}]	Cumulative storage [Mt]	Period	Source Categorisation	Sources
US	EOR	Arkalon	0.29	0.092	Farnsworth Unit	0.092	0.092	0.46	2013-2017	3	54

Table 17: The compiled global geological CCS statistical database for 20 operational commercial-scale CCS facilities between 1996-2020 shows the aggregate 2019 estimates of capture rate capacity, the capture rate, storage rate– hybrid, storage rate– average over individual project lifetime and the cumulative storage. These estimates are compiled using data (black font) from Table 3-16 of the Supporting Information.

Aggregate capture rate Capacity 2019-2020 [Mt yr^{-1}]	Aggregate Capture Rate [Mt yr^{-1}]	Aggregate storage Rate - hybrid [Mt yr^{-1}]	Aggregate storage Rate - average over individual project lifetime [Mt yr^{-1}]	Cumulative storage [Mt]
**35.76**	**31.30**	**28.90**	**25.09**	**196.68**

REFRENCES

1. Shukla, P.R., Skea, J., Slade, R., Al Khourdajie, A., vanDiemen, R., McCollum, D., Pathak, M., Some, S., Vyas, P., Fradera, R., Belkacemi, M., Hasija, A., Lisboa, G., Luz, S., Malley, J., (eds.). *Climate Change 2022: Mitigation of Climate Change. Contribution of Working Group III to the Sixth Assessment Report of the Intergovernmental Panel on Climate Change*; IPCC, 2022. doi: 10.1017/9781009157926
2. Page, B., Turan, G. & Zapantis, A. *Global Status of CCS: 2020, 2020*; Global CCS Institute; https://www.globalccsinstitute.com/wp-content/uploads/2021/03/Global-Status-of-CCS-Report-English.pdf (accessed October 6, 2021).
3. *Carbon Capture and Sequestration Project Database*. Carbon Capture & Sequestration Technologies at MIT. *http://sequestration.mit.edu/tools/projects/index.html* (accessed October 6, 2021).
4. *Carbon Capture and Storage Database.* National Energy Technology Laboratory; https://netl.doe.gov/coal/carbon-storage/worldwide-ccs-database (accessed October 6, 2021).
5. *CCS Database.* ZeroCO2. http://www.zeroco2.no/projects (accessed Octorber 6, 2021).
6. *Global pipeline of commercial CCUS facilities operating and in development, 2010-2021.* International Environmental Agency Website. https://www.iea.org/data-and-statistics/charts/global-pipeline-of-commercial-ccus-facilities-operating-and-in-development-2010-2021 (accessed October 6, 2021).

7. Martin-Roberts, E., Scott, V., Flude, S., Johnson, G., Haszeldine, R.S. & Gilfillan, S. Carbon capture and storage at the end of a lost decade. *One Earth,* 4(11), 1569-1584 (2021). https://doi.org/10.1016/j.oneear.2021.10.002
8. Loria, P. & Bright, M.B.H. Lessons captured from 50 years of CCS projects. *The Electricity Journal,* **34** (7), 196998 (2021).
9. IEA; *About CCUS*; International Environmental Agency: Paris, 2021 https://www.iea.org/reports/about-ccus (accessed October 6, 2021).
10. *National Inventory Submissions 2020.* United Nations Convention on Climate Change Website; *https://unfccc.int/ghg-inventories-annex-i-parties/2020* (accessed October 6, 2021).
11. *EPA Facility Level Information on GreenHouse gases Tool.* U.S. Environmental Protection Agency Website. *https://ghgdata.epa.gov/ghgp/main.do#/facility/?q=Find%20a%20Facility%20or%20Location&st=&bs=&et=&fid=&sf=11001100&lowE=-20000&highE=23000000&g1=1&g2=1&g3=1&g4=1&g5=1&g6=0&g7=1&g8=1&g9=1&g10=1&g11=1&g12=1&s1=1&s2=1&s3=1&s4=1&s5=1&s6=1&s7=1&s8=1&s9=1&s10=1&s201=1&s202=1&s203=1&s204=1&s301=1&s302=1&s303=1&s304=1&s305=1&s306=1&s307=1&s401=1&s402=1&s403=1&s404=1&s405=1&s601=1&s602=1&s701=1&s702=1&s703=1&s704=1&s705=1&s706=1&s707=1&s708=1&s709=1&s710=1&s711=1&s801=1&s802=1&s803=1&s804=1&s805=1&s806=1&s807=1&s808=1&s809=1&s810=1&s901=1&s902=1&s903=1&s904=1&s905=1&s906=1&s907=1&s908=1&s909=1&s910=1&s911=1&si=&ss=&so=0&ds=E&yr=2020&tr=current&cyr=2020&ol=0&sl=0&rs=ALL* (accessed October 6, 2021).
12. *Guidelines for the technical review of information reported under the Convention related to greenhouse gas inventories, biennial reports and national communication by parties included in Annex I to the Convetion*, FCCC/CP/2014/10/Add.3 (General. Feb 2, 2015)
13. *GHGRP methodology and verification.* U.S. Environmental Protection Agency Website. https://www.epa.gov/ghgreporting/ghgrp-methodology-and-verification (accessed October 6, 2021).
14. *GRI mission & history.* Global Reporting Initiative Website. https://www.globalreporting.org/about-gri/mission-history/ (accessed October 6, 2021).
15. *Cai, B., Li, Q., Lin, Q. & Ma, J. China Carbon Capture Utilisation and Storage (CCUS) Report 2019;* Chinese Academy of Environmental Planning; Beijing, 2020.
16. *Oxy 2020 Annual ESG Performance Indicators*. Occidental Petroleum Website. *https://www.oxy.com/globalassets/documents/sustainability/annualperformancesummarytable.pdf* (accessed October 6, 2021).

17. *Information on Geological Sequestration of Carbon Dioxide, Hobbs Field.* U.S. Environmental Protection Agency Website. https://ghgdata.epa.gov/ghgp/service/facilityDetail/2019?id=1012121&ds=A&et=&popup=true (accessed October 6, 2021).

18. *Information on Geological Sequestration of Carbon Dioxide, Denver Unit.* U.S. Environmental Protection Agency Website. https://ghgdata.epa.gov/ghgp/service/facilityDetail/2019?id=1011767&ds=A&et=&popup=true (accessed October 6, 2021).

19. *Sleipner.* Norwegian Petroleum Directorate Website. https://www.norskpetroleum.no/en/facts/field/sleipner-ost/ (accessed October 6, 2021).

20. *Snohvit.* Norwegian Petroleum Directorate Website. https://www.norskpetroleum.no/en/facts/field/snohvit/ (accessed October 6, 2021).

21. *Gorgon project carbon dioxide injection project low emission technology demonstration fund annual report 1 July 2016 - 30 June 2017*; Department of Industry, Innovation and Science Western Australia Government: Western Australia, 2017.

22. *Gorgon project carbon dioxide injection project low emission technology demonstration fund annual report 1 July 2019 – 30 June 2020*; Department of Industry, Innovation and Science Western Australia Government: Western Australia, 2020.

23. *Boundary Dam Carbon Capture Project.* Saskpower Website. https://www.saskpower.com/Our-Power-Future/Infrastructure-Projects/Carbon-Capture-and-Storage/Boundary-Dam-Carbon-Capture-Project (accessed Octorber 6, 2021).

24. *Quest Carbon Capture and Storage Project Annual Summary Report*; Department of Energy: Alberta, 2019a; https://open.alberta.ca/dataset/f74375f3-3c73-4b9c-af2b-ef44e59b7890/resource/ff260985-e616-4d2e-92e0-9b91f5590136/download/energy-quest-annual-summary-alberta-department-of-energy-2019.pdf (accessed Octorber 6, 2021).

25. *Quest CO2 Capture Ratio Performance*; Department of Energy: Alberta, 2019b; http://pubsapp.acs.org/paragonplus/submission/esthag/est-citation-sample.pdf? (accessed Octorber 6, 2021).

26. Pacala, S., & Socolow, R. Stabilization wedges: solving the climate problem for the next 50 years with current technologies. *Science*. 2004, *305*(5686), 968-972. doi: 10.1126/science.1100103

27. *Snapshot of Global PV Markets 2021;* Report IEA-PVPS T1-39:2021; Paris, 2021; https://iea-pvps.org/wp-content/uploads/2021/04/IEA_PVPS_Snapshot_2021-V3.pdf (accessed November 12, 2021).

28. *Statistical Review of World Energy;* BP website;2021 (accessed June 13, 2022)

29. Avoided Emissions Calculator. IRENA website. https://www.irena.org/climatechange/Avoided-Emissions-Calculator (accessed June 9, 2022)
30. Abdulla, A., Hanna, R., Schell, K. R., Babacan, O. & Victor, D. G. Explaining sucessful and failed investments in U.S. carbon capture and storage using empirical and expert assessments. *Enviornmental Research Letters.* **2021**, *16(1).* doi: 10.1088/1748-9326/abd19e
31. Zahasky, C., & Krevor, S. Global geologic carbon storage requirements of climate change mitigation scenarios. *Energy Environment. Sci.* **13,** 1561-1567 (2020). https://doi.org/10.1039/D0EE00674B
32. Zhang, Y., Jackson, C., Zahasky, C., Nadhira, A. & Krevor, S. European carbon storage resource requirements of climate change mitigation targets. *Int. J. Greenh. Gas Control* **114,** 103568 (2022). https://doi.org/10.1016/j.ijggc.2021.103568
33. Ringrose, P. S., & Meckel, T. A. Maturing global CO2 storage resources on offshore continental margins to achieve 2DS emissions reductions. *Scientific reports*, **9**(1), 1-10 (2019).
34. Martin-Roberts, E., Scott, V., Flude, S., Johnson, G., Haszeldine, R. S., & Gilfillan, S. Carbon capture and storage at the end of a lost decade. *One Earth*, **4**(11), 1569-1584 (2021). https://doi.org/10.1016/j.oneear.2021.10.002
35. Petrobras. *ESG.* Petrobras: Rio de Janeiro 2020a. https://api.mziq.com/mzfilemanager/v2/d/25fdf098-34f5-4608-b7fa-17d60b2de47d/2a0029b5-75e7-ad89-7c3e-30793604118e?origin=1 (accessed November 12, 2021).
36. Petrobras. *Sustainability report 2019*. Petrobras: Rio de Janeiro, 2020b. https://sustentabilidade.petrobras.com.br/en/src/assets/pdf/Sustainability-Report.pdf (accessed November 12, 2021).
37. *Our 2020 Sustainability report advances in 2020.* Petrobras Website. *https://petrobras.com.br/en/news/our-2020-sustainability-report-with-advances-in-esg.htm* (accessed November 12, 2021).
38. Petrobras. Sustainability report 2020. Petrobras: Rio de Janeiro, 2020c https://sustentabilidade.petrobras.com.br/en/ (accessed November 12, 2021).
39. Petroleum Technology Research Centre. Innovation, Alliances & Sustainability 2019 – 2020 Annual Report. Petroleum Technology Research Centr, 2020. https://ptrc.ca/pub/docs/annual-reports/Annual%20Report_Shortened.pdf (accessed November 12, 2021).
40. *BD3 Status Update: May 2021.* Saskpower Website https://www.saskpower.com/about-us/our-company/blog/2021/bd3-status-update-may-2021 (accessed November 12, 2021).

41. *Corporate Presentation.* Whitecap Resources Website. https://www.wcap.ca/application/files/3316/2403/8674/WCP_2021_06_18.pdf (accessed November 12, 2021).
42. *Eastern Saskatchenwan Business Unit.* Whitecap Resources Website. https://www.wcap.ca/operations/core-areas/southeast-saskatchewan (accessed November 12, 2021).
43. *CO2 Sequestration White Resources A Responsible Energy.* Whitecap Resources Website. *Story*https://www.wcap.ca/sustainability/co2-sequestration (accessed November 12, 2021).
44. *Carbon capture milestone reached at Dakota Gas.* Dakota Gas Website. https://dakotagas.com/News-Center/news-releases/carbon-capture-milestone-reached-at-dakota-gas (accessed November 12, 2021).
45. *Quest CCS facility capture and stores five million tonnes of CO2 ahead of fifth anniversary.* Shell Website. https://www.shell.ca/en_ca/media/news-and-media-releases/news-releases-2020/quest-ccs-facility-captures-and-stores-five-million-tonnes.html (accessed November 12, 2021).
46. Shell. *Sustainability report 2020*. Royal Dutch Shell: London, 2021 *https://reports.shell.com/sustainability-report/2020/servicepages/downloads/files/shell-sustainability-report-2020.pdf* (accessed October 6, 2021).
47. 2021-03-10 MP Blaine Calkins Member Statement. *Enhance Energy*. Vimeo, March 10, 2021. https://vimeo.com/527500313 (accessed November 1, 2021)
48. *Megatonne Milestone – Charting a new and cleaner path forward by capturing CO2 and producing lower carbon energy.* Enhance Energy Website. https://enhanceenergy.com/megatonne-milestone-charting-a-new-and-cleaner-path-forward-by-capturing-co2-and-producing-lower-carbon-energy/ (accessed March 7, 2022).
49. China National Petroleum Corporation. *Corporate Social Responsibility Report*. China National Petroleum Corporation: Beijing, 2021 https://www.cnpc.com.cn/en/csr2020enhmshn/202105/64f93c5684754f859b9b81602fba1979/files/e52454722e1c4c269071c16987f70255.pdf (accessed October 6, 2021)
50. Norwegian Environment Agency. *Greenhouse Gas Emissions 1990-2019 National Inventory Report*. Norwegian Environment Agency: Oslo, 2021. https://unfccc.int/documents/273425 (accessed October 6, 2021).

51. Equinor. *Sustainability Report 2020*. Equinor: Stavanger, 2021 https://www.equinor.com/en/investors/annual-reports.html#downloads (accessed October 6, 2021).

52. *2019 Information on Geological Sequestration of Carbon Dioxide*; Archer Daniels Midland Co. U.S. Environmental Protection Agency Website. https://ghgdata.epa.gov/ghgp/service/facilityDetail/2019?id=1005661&ds=E&et=&popup=true (accessed October 6, 2021).

53. Archer Daniels Midland. *2020 Corporate Sustainability Report*. Archer Daniels Midland: Chicago, Ilinois, 2021 https://assets.adm.com/Sustainability/3860041_20_Archer-Daniels-Midland_ESG-Report_WR.pdf (accessed October 6, 2021).

54. National Energy Technology Laboratory. *Farnsworth Unit Project*. National Energy Technology Laboratory, 2017. https://www.netl.doe.gov/sites/default/files/2018-11/Farnsworth-Unit-Project.pdf (accessed October 6, 2021).

55. U.S. Environmental Protection Agency. *Technical Review of Subpart RR MRV Plan for North Burbank Unit.* U.S. Environmental Protection Agency, 2020. https://www.epa.gov/sites/default/files/2020-12/documents/nbu_decision.pdf (accessed October 6, 2021).

56. Exxon Mobile. *Updated 2021 Energy & Carbon Summary.* Exxon Mobile: Irving, Texas, 2021. https://corporate.exxonmobil.com/-/media/Global/Files/energy-and-carbon-summary/Energy-and-Carbon-Summary.pdf (accessed October 6, 2021).

57. Chevron. *Climate Change Resilience Report.* Chevron: San Ramon, California, 2021. https://www.chevron.com/-/media/chevron/sustainability/documents/climate-change-resilience-report.pdf (accessed September 16, 2021).

58. Evens, D. Chevron filas to hit targets with giant CCS scheme at Gorgon LNG. *Energy Voice (Aberdeen)*, July 20, 2021. https://www.energyvoice.com/oilandgas/asia/337852/chevron-fails-to-hit-targets-with-giant-ccs-scheme-at-gorgon-lng/#:~:text=the%20WhatsApp%20logo.-,Chevron%20fails%20to%20hit%20targets%20with%20giant%20CCS%20scheme%20at,LNG)%20export%20project%20in%20Australia (accessed September 16, 2021).

59. Denbury. *2021 Corporate Responsibility Report.* Denbury: Plano, Texas, 2021. https://s27.q4cdn.com/166477028/files/doc_downloads/Denbury-2021-Corporate-Responsibility-Report.pdf (accessed October 6, 2021).

60. *Denbury 26th Annual Credit Suisse Energy Summit. https://s1.q4cdn.com/594864049/files/doc_downloads/2021/03/03-2021-Credit-Suisse-Presentation.pdf* (accessed October 6, 2021).

61. *Information on Geological Sequestration of Carbon Dioxide, Core Energy Otsego County EOR Operations.* U.S. Environmental Protection Agency Website. https://ghgdata.epa.gov/ghgp/service/facilityDetail/2020?id=1010117&ds=A&et=&popup=true (accessed July 8, 2022).
62. *Key Performance.* Sinopec Website. http://www.sinopec.com/listco/en/csr/sustainability/ydqhbh.shtml (accessed Octorber 6, 2021).
